# Prospects of adjuvant RANKL inhibition in breast cancer?

**DOI:** 10.1038/cddis.2015.332

**Published:** 2015-11-19

**Authors:** T D Rachner, P Wimberger, L C Hofbauer

**Affiliations:** 1 Division of Endocrinology and Metabolic Bone Diseases, Department of Medicine III, Technical University, Dresden, Germany; 2 Department of Gynecology and Obstetrics, Technical University, Dresden, Germany; 3 Center for Regenerative Therapies, Technical University, Dresden, Germany

The maintenance of bone health has been a clinical challenge in breast cancer patients receiving adjuvant endocrine treatment. Especially in the setting of hormone receptor-positive breast cancer, treatment with aromatase inhibitors, although highly effective as anti-cancer agents, causes rapid and substantial bone loss.^1^ In the bone environment, receptor activator of nuclear factor-*κ*B ligand (RANKL) is an essential determinant of osteoclast differentiation and activity. Denosumab is a monoclonal antibody directed against RANKL that has been approved for the treatment of both osteoporosis and bone metastases.^1^ Although an earlier study had shown a significant increase of 5.5% in bone mineral density (BMD) at the lumbar spine with denosumab in 252 women with non-metastatic breast cancer receiving aromatase inhibitors,^2^ this trial was not designed to assess fracture risk.

In a recent study, Gnant *et al.*^3^ reported results of the ABCSG-18 trial. This multicenter trial assessed the use of denosumab for fracture prevention in postmenopausal women with estrogen receptor-positive breast cancer receiving an aromatase inhibitor treatment. More than 3000 patients were enrolled to receive either denosumab at the standard osteoporosis dose of 60 mg twice yearly or placebo. In the denosumab group, the time to first fracture was significantly delayed by 50% (HR 0.5; 95% CI 0.39–0.65). Notably, a similar fracture reduction was seen independent of the initial *T*-score of the BMD. There was no difference in adverse events between the placebo and the denosumab group. In fact, most adverse events were considered to be aromatase inhibitor related. No cases of osteonecrosis of the jaw or atypical fractures were reported in either group.^3^ These findings are important as they clearly demonstrate a substantial clinical benefit with the use of denosumab in the adjuvant treatment of hormone-positive breast cancer, while displaying a favorable safety profile.

In the past years, emerging preclinical and clinical findings have corroborated a role of the RANKL–RANK system in the pathophysiology of breast cancer exceeding that of being a simple osteoclast differentiation factor (Figure 1). RANKL has been proposed to be a key mediator of progestin-driven mammary carcinogenesis.^4, 5^ Administration of the synthetic progesterone derivate medroxyprogesterone acetate (MPA) and the carcinogen 7,12-dimethylbenz[a]anthracene results in an enhanced carcinogenesis, which is driven by a massive increase of RANKL. Genetic inhibition of RANK markedly reduced the incidence of cancer in this setting.^4^ Similar results were obtained in another study, where pharmacological inhibition of RANKL attenuated mammary tumor development.^5^

Furthermore, RANKL has been directly linked to the occurrence of bone metastases by increasing the migration of various malignant cells including breast, prostate and melanoma by binding its receptor RANK.^6, 7^ In a preclinical model of melanoma, neutralization RANKL by its decoy receptor osteoprotegerin markedly reduced bone metastases.^6^ In addition, mammary cancer metastasis to the lung have been shown to be promoted by the presence of tumor-infiltrating regulatory T cells, which produce high levels of RANKL.^8^

Indeed, expression levels of RANKL and RANK are increased in metastatic prostate cancer samples (44% and 49%) compared with primary prostate cancer samples (31% and 38%), respectively.^9^ In breast cancer, RANKL expression was observed in 24/40 samples,^10^ whereas RANK overexpression was found in 39% of ductal and 53% of lobular breast carcinomas.^11^

Several studies have assessed the prognostic value of RANK expression in breast cancer patients regarding survival and the propensity for bone metastases.^11, 12, 13^ Low levels of RANK and high levels of osteoprotegerin were correlated with a longer overall survival in microarray analyses.^11^ This was confirmed by two different studies that immunohistochemically evaluated 185 and ~600 breast cancer samples for RANK expression and showed a significant association between RANK and poor disease-free survival.^12, 13^ In addition, RANK has been positively correlated with the development of bone metastases.^11^

The occurrence of bone metastases in the ABCSG-18 trial was too low to assess a benefit of denosumab, which was expected considering the low-recurrence risk in this cohort. However, another trial entitled D-CARE (NCT01077154) is currently underway to specifically address the question whether denosumab treatment can delay the occurrence of bone metastases in patients who are at a higher risk of recurrence than those of the ABCSG-18 trial. The cohort included in this trial has a higher risk of bone recurrence and will provide further insights on the extend of clinical benefit of adjuvant RANKL inhibition in breast cancer patients.

In summary, the results from the ABCSG-18 trial show a benefit for patients with hormone-positive breast cancer receiving adjuvant denosumab in terms of fracture reduction while on aromatase inhibitors. These findings are in line with an increasing evidence to suggest that RANKL and RANK may have disease modifying effects in breast cancer by affecting carcinogenesis and the process of bone metastases.

## Figures and Tables

**Figure 1:**
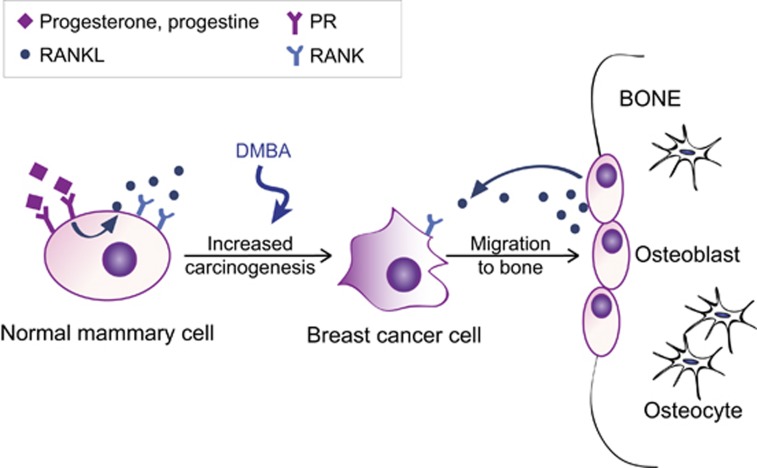
Impact of RANK/RANKL signaling on breast cancer. Progesterone receptor signaling in breast tissue results in a strong upregulation of RANKL expression. This is considered to mediate progestin-driven mammary carcinogenesis. Bone-derived RANKL promotes the migration of RANK expressing breast cancer cells to bone.
